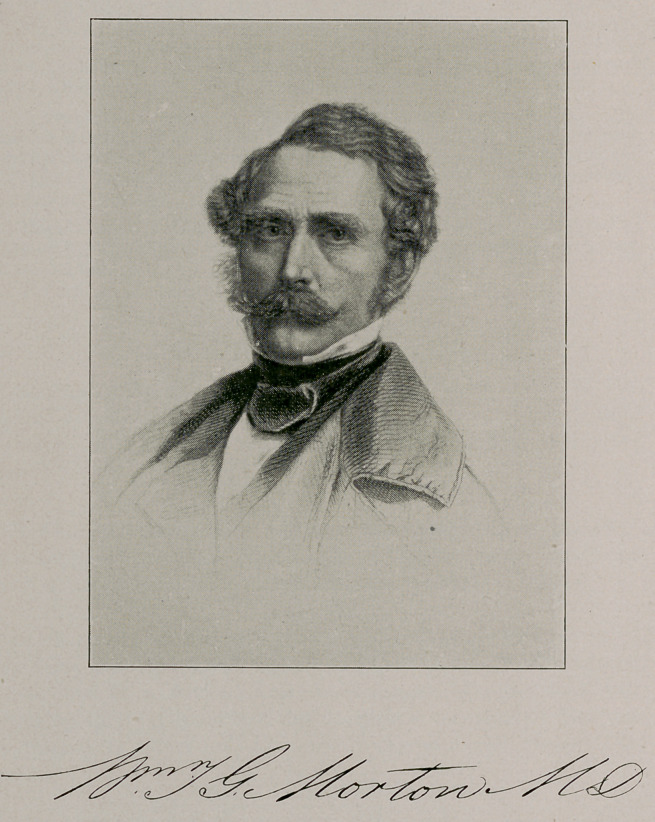# Dr. William T. G. Morton in Buffalo

**Published:** 1896-11

**Authors:** 


### DR. WILLIAM T. G. MORTON IN BUFFALO.

IN CONNECTION with the semi-centennial anniversary of anesthesia and the proceedings at Buffalo University Medical College, we extract the following from the records of

### THE MEDICAL SOCIETY OF THE COUNTY OF ERIE.

*Special Meeting, held Saturday, February 4, 1865.*

A special meeting of the Medical Society of the County of Erie was held at their new rooms, in the Young Men's Association building, Saturday, February 4, 1865, at 8 o'clock P. M., for the purpose of hearing some remarks by Dr. William T. G. Morton, on The origin and use of practical anesthesia. The president, Dr. C. C. F. Gay, introduced Dr. Morton to the society, who proceeded to give a detailed account of the discovery by himself of anesthesia and its use in hospitals and on the battlefield.

Upon the conclusion of his remarks, Dr. ROCHESTER addressed the meeting, saying that a testimonial should be given to Dr.

Morton for his discovery, and moved a committee of eight be appointed to take action in the matter, which was adopted.

The PRESIDENT appointed as such committee, Drs. Rochester, C. W. Harvey, Samo, Lothrop, Barnes, C. C. F. Gay, Burwell and Miner.

Upon motion, the society adjourned till Monday, at 8 o'clock P. M., for the further consideration of the subject.

ADJOURNED MEETING, MONDAY, FEBRUARY 6, 1865.

Dr. C. C. F. GAY, president, in the chair.

The committee appointed at the meeting Saturday evening made a report, offering the following resolutions, which were adopted :

The committee appointed to express in a proper manner the thanks of this society to Dr. William T. G. Morton for his full and satisfactory account of his discovery of the anesthetic properties of sulphuric ether and his statement of the difficulties attending its introduction and use, submits the following report:

It appears beyond a doubt to medical men that Dr. Morton is the discoverer of the anesthetic powers of sulphuric ether, and that he was the first to clearly demonstrate that the condition of anesthesia, a state in which there is entire insensibility to pain, could be safely and certainly produced. He is therefore the practical originator of anesthetic inhalation.

Moreover, we are indebted very greatly, if not wholly, to his personal efforts, not only for its discovery, but for its adoption and use; for this precious gift to humanity met at first with incredulity, if not positive hostility, and no ordinary courage and energy were requisite to establish the use of an agent which has been of such incalculable benefit to humanity in preventing and suspending pain. And we may assume that its benefits are not confined to its being the most certain and safe means of alleviating pain yet made use of in surgical practice, for its very power to prevent pain has given increased success to severe operations in surgery.

It further appears that for this important discovery, taking rank among the most important gifts to humanity, analogous to that of the great Jenner, Dr. Morton has not only not received adequate compensation, but has, in his efforts to secure its adoption, made great pecuniary sacrifices, for which he has repeatedly sought, but has yet failed to receive, a natural reward—such a reward as has been in discoveries of like nature, but of less importance, given by European governments.

The great value of the anesthetic inhalation is now fully established. Thousands of painless operations have fully demonstrated its benefits and its safety. It has robbed surgery of its terrors. A great amount of human suffering, which once seemed inevitable, is by its powers wholly prevented. As a profession we daily witness its power and its benefits. We are, therefore, daily reminded of him who first demonstrated the safety and practicability of anesthetic inhalation and established its practice. To him we owe this great benefit, and to him is due the warmest gratitude of mankind. We therefore recommend the passage of the following resolutions:

*Resolved,* That the thanks of the society are hereby tendered to Dr. Morton for his clear and satisfactory statement, in which he has placed beyond doubt his claims as the originator and introducer of anesthetic inhalation.

*Resolved,* That inasmuch as he has failed of national recompense, we commend this appeal to the public to compensate him for the sacrifices he has made in establishing his claims, and especially we urge upon this community to contribute to the testimonial, in order that a most beneficent discovery may not become a pecuniary loss to the discoverer.

The committee was empowered to sign a call inviting the citizens of Buffalo to hear Dr. Morton give an account of the discovery and introduction of ether and chloroform and their use in hospital and army practice as witnessed by himself.

On motion the society adjourned.

LEON F. HARVEY, *Secretary.*

The foregoing is deemed of sufficient interest to warrant its publication at this time. The proceedings of these meetings have never before been printed and ought to be preserved as a part of —indeed, an interesting chapter in—the history of anesthesia.

## Figures and Tables

**Figure f1:**